# 

**Published:** 1878-02

**Authors:** 


### Contents of February Number.

### ORIGINAL.

ART. I.—A case of Intussusception in which Laparotomy was performed. By A. A. Hubbell, M. D., Leon, N. Y., ........... 241
ART. II.—Clinical Lecture upon Plaster of Paris Jacket. By Prof. Julius F. Miner, M. D., .................................... 254

### CORRESPONDENCE.

Fracture of the Shaft of Femur in a Child. By S. J. Radcliffe, M.D., . 255
A few words of Explanation from Prof. James P. White, M. D., ..... 257

### MISCELLANEOUS.

Treatment of Hœmoptysis from Lung Cavities, ........................ 258
The Poison of Typhoid Fever conveyed by Water, .................... 258
Early Rupture of the Membranes in Midwifery, ....................... 259
Therapeutic Action of Balsam of Peru, ............................. 260
Is the Human Eye changing its Form? ............................... 261
The Ætiology of Diphtheria, ....................................... 262
Ozone, ............................................................. 262
Extirpation of the Kidney, ........................................ 263
Children's disorders among the Japanese, .......................... 264
Sclerotic Acid, .................................................... 264
Administration of Salicylic Acid, ................................. 264
Nursing in Columbia, .............................................. 265
Dilatation of the Urethra by the Urine itself, .................... 265
Medical Certificates in the Daily Papers, ......................... 266
Code of Ethics, ................................................... 267
Diphtheria, ....................................................... 270
Intelligent and Efficient Quarantine, ............................. 270
Gratuitous Hint for Advertising Doctors, .......................... 271
Signification of Oxygen, .......................................... 272
Louisville Medical News, .......................................... 272
Detroit Lancet, ................................................... 272

### EDITORIAL.

New Dispensaries, ................................................. 272
Rush Medical College, ............................................. 274
Cronyn's Boudoir Medicines, ....................................... 274
OBITUARY.—Dr. Henry W. Dean, ...................................... 275
BOOKS REVIEWED:
Transactions of American Medical Congress, ........................ 276
Pathological Diagnosis. Orth, ..................................... 279
Books and Pamphlets Received, ..................................... 280

